# The salamander limb: a perfect model to understand imperfect integration during skeletal regeneration

**DOI:** 10.1242/bio.060152

**Published:** 2024-02-06

**Authors:** Camilo Riquelme-Guzmán, Tatiana Sandoval-Guzmán

**Affiliations:** ^1^ Department of Internal Medicine 3, Center for Healthy Aging, University Hospital Carl Gustav Carus at the Technische Universität Dresden, 01307 Dresden, Germany; ^2^ Paul Langerhans Institute Dresden of Helmholtz Centre Munich, University Hospital Carl Gustav Carus at the Technische Universität Dresden, 01307 Dresden, Germany

**Keywords:** Axolotl, Ossification, Bone, Cartilage, Regeneration, Integration

## Abstract

Limb regeneration in salamanders is achieved by a complex coordination of various biological processes and requires the proper integration of new tissue with old. Among the tissues found inside the limb, the skeleton is the most prominent component, which serves as a scaffold and provides support for locomotion in the animal. Throughout the years, researchers have studied the regeneration of the appendicular skeleton in salamanders both after limb amputation and as a result of fracture healing. The final outcome has been widely seen as a faithful re-establishment of the skeletal elements, characterised by a seamless integration into the mature tissue. The process of skeletal integration, however, is not well understood, and several works have recently provided evidence of commonly occurring flawed regenerates. In this Review, we take the reader on a journey through the course of bone formation and regeneration in salamanders, laying down a foundation for critically examining the mechanisms behind skeletal integration. Integration is a phenomenon that could be influenced at various steps of regeneration, and hence, we assess the current knowledge in the field and discuss how early events, such as tissue histolysis and patterning, influence the faithful regeneration of the appendicular skeleton.

## Teaser

**Summary:** This Review explores the progress and new advances in the salamander skeletal biology field, focusing on how the limb skeleton is efficiently regenerated upon amputation or injury.

## Introduction

Salamanders (urodeles) are a diverse group in their life cycle and regeneration capabilities (Brockes, 2015). The axolotl (*Ambystoma mexicanum*) and the Spanish newt (*Pleurodeles waltl*) are two of the best-studied species and robust models for limb regeneration (Joven et al., 2019). A key hallmark of this process is the generation of a blastema, a heterogeneous cell mass formed at the amputation plane that mainly contains progenitor cells. In a feedback loop with nerve signals, these populations orchestrate the regeneration of the missing structure (Tanaka, 2016). In the last few decades, the generation of transgenic lines (Fei et al., 2018; Hayashi et al., 2013; Khattak et al., 2013), the sequencing of their genomes (Elewa et al., 2017; Nowoshilow et al., 2018) and other technological advancements have allowed researchers to further use these model organisms to disentangle the cellular and molecular mechanisms governing limb regeneration.

**Fig. 1.**
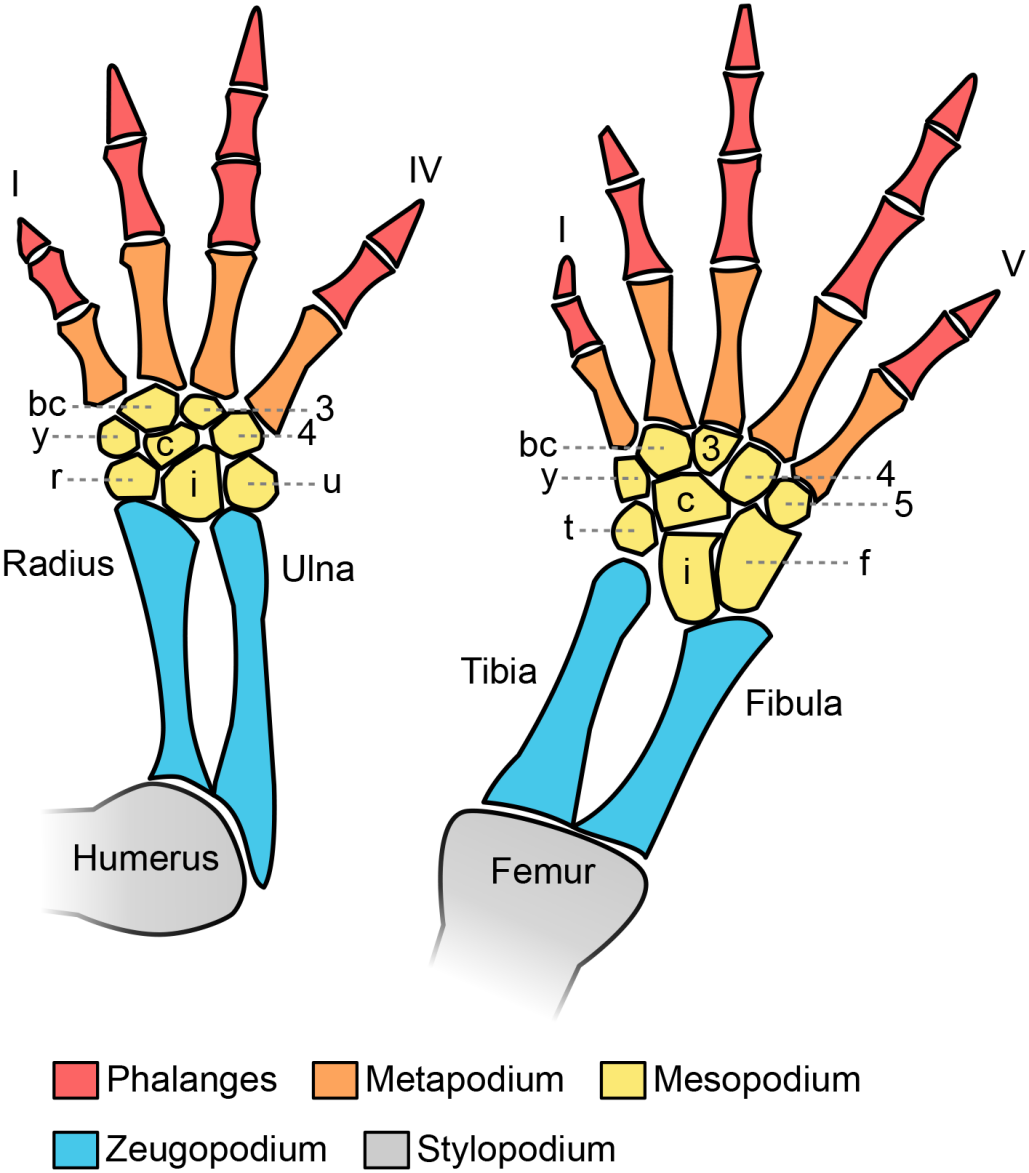
**Salamander appendicular skeleton: A generalised morphology of the appendicular skeleton is shown based on genus Dicamptodon, which is adapted from** **Shubin and Wake (2003****).** Forelimbs present four digits and hindlimbs, five digits. Roman numbers depict the digit number from anterior to posterior. Mesopodium elements are: bc, basal commune; c, centrale; y, element y (centralia 1 in Bothe et al., 2021); r/t, radiale/tibiale; i, intermedium; 3, distal carpal/tarsal 3; 4, distal carpal/tarsal 4; 5, distal tarsal 5; u/f, ulnare/fibulare.

Structurally, the salamander limb resembles that of humans, providing a suitable frame for comparative studies. Although some variations among the different living taxa are evident, a generalised morphology was proposed for the purpose of comparison (Fig. 1) (Shubin and Wake, 2003): Forelimbs and hindlimbs have a stylopodium (humerus and femur), a zeugopodium (radius – ulna and tibia – fibula) and an autopodium (*manus* and *pes*) with no more than four and five digits, respectively. The variability among species is observed in the autopodium, particularly in the number of mesopodium elements, and a limited variation in the number of phalanges in the digits.

The skeleton is the most prominent tissue in the salamander limb, and several publications have addressed the mechanisms involved in its ossification and regeneration. The latter has been shown to resemble development in some respects, proving the importance of understanding both processes for drawing correct conclusions and broaden our knowledge of salamander biology. In this Review, we provide an insight into the current knowledge of salamander skeletal biology, with a particular focus on skeletal regeneration and integration. We present an overview of ossification of the limb skeleton as a ground for discussing the process of skeletal regeneration. Moreover, salamanders have proven useful for studying skeletal-specific injuries, such as fractures, and thus we examine the current advances in this field. A key aspect of successful regeneration is the correct and seamless integration of the regenerate, i.e. a proper amalgamation of the newly-formed skeleton at the amputation plane, which allows for a complete restoration of the limb function. Here we discuss what it is known about integration and what are the challenges laying ahead to unravel the mechanisms regulating it. Finally, we provide an outlook of the new research opportunities in this field.

## How are bones formed in salamanders?

In salamanders, the sequence of chondrification and ossification varies among species, attributed to the different reproductive strategies and environmental backgrounds (Fröbisch, 2008; Harrington et al., 2013). In mammals, limb skeleton develops via endochondral ossification (thoroughly reviewed in Kozhemyakina et al., 2015; Olsen et al., 2000). During this process, the initial formation of a skeletal scaffold is achieved by the condensation of mesenchymal progenitors forming a cartilage primordium. Cells within this primordium are round immature chondrocytes. Eventually those lying in the central regions mature and differentiate, increasing their volumes, becoming hypertrophic chondrocytes. The cycles of proliferation and differentiation of chondrocytes lead to a longitudinal growth of the cartilaginous skeletal elements from the epiphysis (rounded end) towards the diaphysis (midshaft). A pivotal moment during endochondral ossification is the recruitment of blood vessels, osteoclasts and osteoblasts towards the diaphysis. As a result, the cartilage matrix is degraded and osteoblast differentiation results in the deposition of bone (i.e. ossification).

**Fig. 2.**
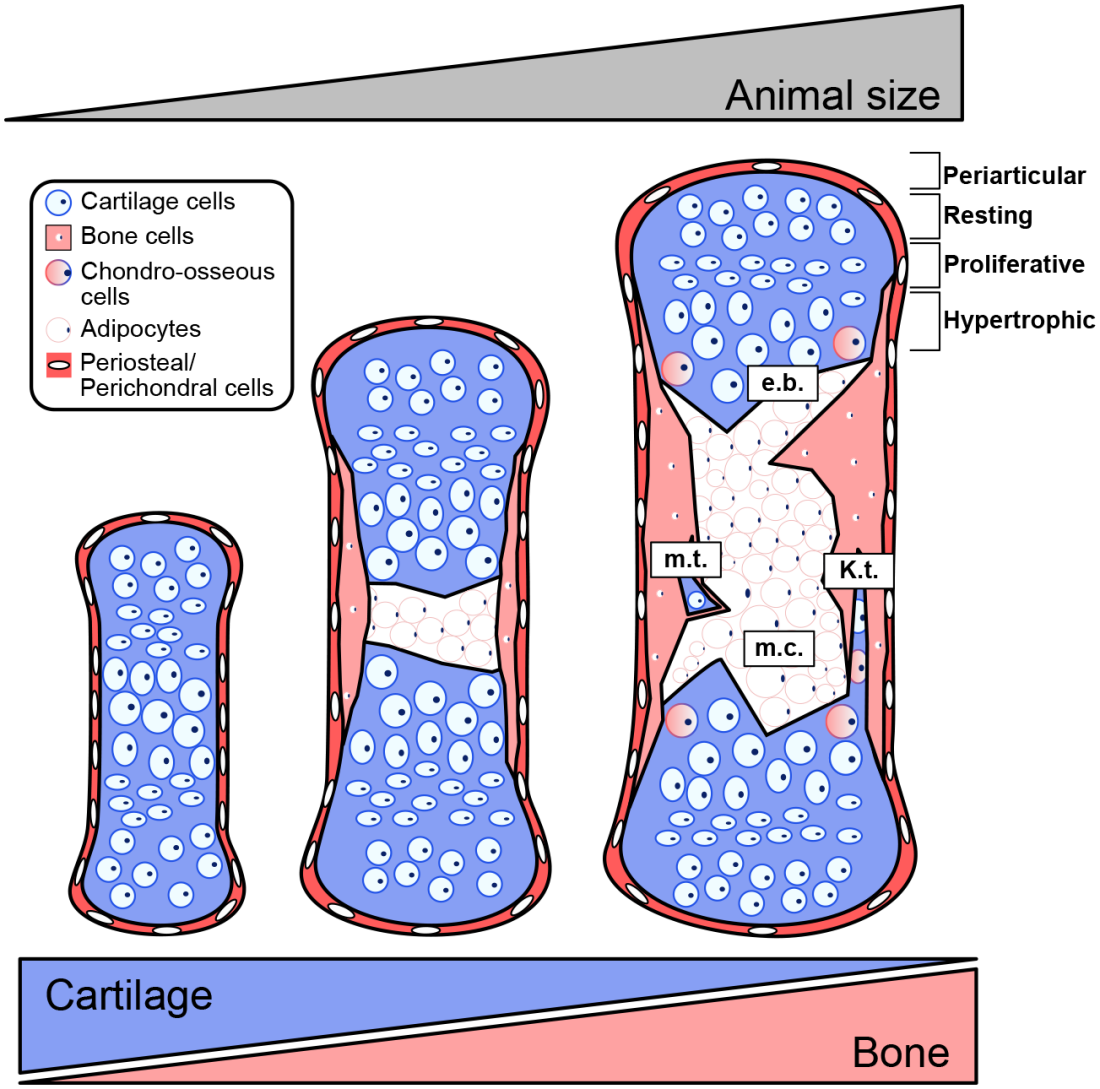
**Ossification of appendicular long bones: Post-embryonic ossification of the limb in salamanders involves an endochondral and periosteal ossification, degradation of cartilage anlage and formation of a marrow cavity (m.c.) filled with adipocytes.** Ossification has been associated with an animal's size; however, elements are not fully ossified as epiphyseal cartilage remains throughout the salamander life cycle. The participation of hypertrophic chondrocytes in ossification has been proposed and the presence of hypertrophic chondrocytes expressing osteogenic markers has been demonstrated (Chondro-osseous cells). Finally, the cartilage presents a loose organisation, but various zones can be identified, i.e. periarticular, resting, proliferative and hypertrophic zones. Other features of salamander appendicular elements include the erosion bay (e.b.), medullary trabeculae (m.t.) and Katschenko's line (K.t.).

In axolotls, ossification in limbs starts in juveniles that are close to present secondary sexual characteristics (Riquelme-Guzmán et al., 2021), while in the newt, it initiates in late active larval stages, shortly after the completion of limb development and before metamorphosis (Kaucka et al., 2022). Ossification starts along the diaphysis with deposition of cortical bone, which in axolotls is accompanied by cartilage remodelling, vascularisation and formation of a marrow cavity filled with adipocytes and devoid of a hematopoietic niche (Fig. 2) (Cosden-Decker et al., 2012; Lopez et al., 2014; Polikarpova et al., 2022; Riquelme-Guzmán et al., 2021). It is important to note that the acellular calcified ring surrounding the cartilaginous diaphysis is replaced by ossified tissue, a mechanism driven by osteoblasts (Riquelme-Guzmán et al., 2021).

In contrast, the initial cortical bone deposition in the Spanish newt is uncoupled from cartilage degradation (Kaucka et al., 2022). This initial ossification along the diaphysis corresponds to a distinct process of periosteal ossification (intramembranous ossification) (Castanet et al., 2003; Kaucka et al., 2022), which involves the direct formation of bone by the perichondrium. In newts, cortical bone grows along the periphery of the skeletal element, towards the epiphyses by an ossification notch. Simultaneously, the cartilage keeps growing due to chondrocyte proliferation and hypertrophy (Ricqlès, 1964, 1965). Closer to metamorphosis, erosion of the cartilage matrix and deposition of bone starts (endochondral ossification), which is coupled with the formation of a marrow cavity (Kaucka et al., 2022; Ricqlès, 1964, 1965).

The formation of the marrow cavity is initiated by resorbing cells, occurring in so called ‘erosion bays’, which expand longitudinally in the direction of the epiphyses (Quilhac et al., 2014). These bays are of variable size, resulting in irregular cavities, leaving behind ‘medullary trabeculae’, i.e. patches of ossified cartilage (Castanet et al., 2003; Quilhac et al., 2014). Interestingly, paedomorphic salamanders (those that retain larval features throughout their lives) keep a thin cartilaginous layer along the cortical bone, which is sometimes referred to as ‘Katschenko's line’ (Castanet et al., 2003). This cartilaginous layer is found in older axolotl specimens (>5-years-old) (Riquelme-Guzmán et al., 2021), and has been used as feature to assess paedomorphism in fossil records (Sanchez et al., 2010; Skutschas and Stein, 2015). Comparatively, in salamanders that undergo metamorphosis and have a terrestrial habitat (newts), ossification of the diaphysis is complete in fully mature adults (Castanet et al., 2003).

Even though the diaphysis is completely ossified, no secondary ossification centre is formed in salamanders, and the epiphyses remain mostly cartilaginous (Haines, 1942; Molnar, 2021; Riquelme-Guzmán et al., 2021; Rux et al., 2018). Structurally, epiphyses can adopt two conformations: a concave epiphysis, which extends towards the mid-diaphysis, or a convex epiphysis, which is a cartilaginous cap separated from the marrow cavity (Molnar, 2021). The lack of epiphyseal ossification was proposed to be an adaptation to the life cycle of salamanders (Castanet et al., 2003; Haines, 1942) and likely a mechanism contributing to the longitudinal growth of long bones (Cosden-Decker et al., 2012; Hanken, 1982). The epiphyseal cartilage (and also the cartilage anlage before ossification) presents a loose cellular organisation when compared to mammals (Haines, 1942; Molnar, 2021; Riquelme-Guzmán et al., 2021), which is a consequence of the orientation of chondrocytes expansion (Kaucka et al., 2022). Nevertheless, it is possible to identify the different regions commonly associated with this tissue; namely the existence of periarticular, resting, proliferative, pre-hypertrophic and hypertrophic zones (Polikarpova et al., 2022; Quilhac et al., 2014). Interestingly, evidence suggests a higher proportion of epiphyseal cartilage in aquatic salamanders compared to terrestrial ones (Molnar, 2021). Unfortunately, it remains unknown how variations on epiphyseal cartilage are influenced by other relevant life hallmarks, such as sexual maturity and aging. In the long-lived axolotl, an expansion of the marrow cavity towards the epiphysis was reported in 10-year-old animals (Rux et al., 2018) and periosteal ossification of the epiphysis occurred in one 20-year-old specimen (Riquelme-Guzmán et al., 2021), suggesting a dynamic and continuous ossification of the long bones throughout their lives.

The last elements to ossify are the ones in the mesopodium (carpals and tarsals). These elements ossify late during post-embryonic development or remain cartilaginous for the entire life (Fröbisch, 2008; Jia et al., 2022). In axolotls, a variable ossification of these elements was reported in animals older than 5-years-old (Riquelme-Guzmán et al., 2021).

### Metamorphosis influence on ossification

Metamorphosis is a critical event during the biphasic life of many salamanders, producing important body transformations that allow the animal to adapt to new environmental conditions. The changes observed during metamorphosis are a result of the tissue sensitivities to thyroid hormones (TH); triiodothyronine (T_3_) and thyroxine (T_4_). The levels of these hormones increase closer to metamorphosis and reach their peaks at the height of the process, after which TH concentrations decrease to basal levels similar to the ones observed in larval stages (Alberch et al., 1986; Larras-Regard et al., 1981). A connection between TH and the skeleton has been shown in salamanders, particularly for the skull (Rose, 2021; Vassilieva and Smirnov, 2021); however, evidence for the appendicular skeleton remains scarce. To date, most of it comes from the facultative paedomorphic axolotl.

Axolotls do not undergo metamorphosis naturally; however, it can be induced by administration of T_3_ or T_4_ (Khattak et al., 2014). This has allowed researchers to evaluate and understand the mechanisms behind TH influence on ossification. Inducing metamorphosis in axolotls accelerates ossification of the zeugopodium elements (Riquelme-Guzmán et al., 2021), while also possibly reducing their length (Thampi et al., 2018) when compared to paedomorphic siblings. The acceleration of the post-embryonic development in TH-treated axolotls does not only affect the skeletal elements, but the whole limb (Brown, 1997). Remarkably, studies in the newt skull (Smirnov et al., 2020) suggest that tissue sensitivities to TH might depend on the developmental time and be different for each specific skeletal element.

It is noteworthy that salamanders are a group that encompasses individuals with different life cycles. These variations include direct developers and paedomorphosis (facultative and obligate) and it remains unclear what influence TH has in these species and their skeletons (Laudet, 2011; Vassilieva and Smirnov, 2021). Although some salamanders do not undergo metamorphosis, functional TH receptors are present in the axolotl (Safi et al., 2004), which would render the appendicular skeleton sensitive to TH treatments. However, whether endogenous levels of TH play a role at all in the appendicular skeleton of non-metamorphic salamanders remains to be investigated. All in all, the existence of different life history modes emphasises the need to evaluate the development and ossification of the appendicular skeleton in light of the ecological background and behaviour of each individual species (Rose, 2021).

### Cell biology of the appendicular skeleton

Many cartilage- and bone-related markers have been identified in salamander skeleton (Table 1). A key regulator of cartilage formation and chondrocyte differentiation is the transcription factor SOX9 (Bi et al., 1999, 2001), which is broadly present in salamander's cartilage (Kaucka et al., 2022; Riquelme-Guzmán et al., 2021). The ECM component COL2A1 has also been observed in this tissue (Cosden-Decker et al., 2012; Kaucka et al., 2022; Khattak et al., 2013). Additionally, all skeletal cells in cartilage and bone were found to be COL1A2^+^, both by the expression of a reporter induced by *Col1a2* promoter and immunofluorescence (Gerber et al., 2018; Riquelme-Guzmán et al., 2021). Another critical component for cartilage development is the *PTHrP*/*Ihh* signalling pathway, which presents a similar profile in salamanders as in other models. Specifically, *PTHrP* is expressed in the periarticular and resting zones with a moderate expression in the proliferative zone, while *Ihh* expression was observed in pre-hypertrophic and early hypertrophic chondrocytes (Kaucka et al., 2022). On the other hand, in the ossified regions, only the expression of osteocalcin and collagen type I has been reported (Mitogawa et al., 2015; Riquelme-Guzmán et al., 2021).

**Table 1.**
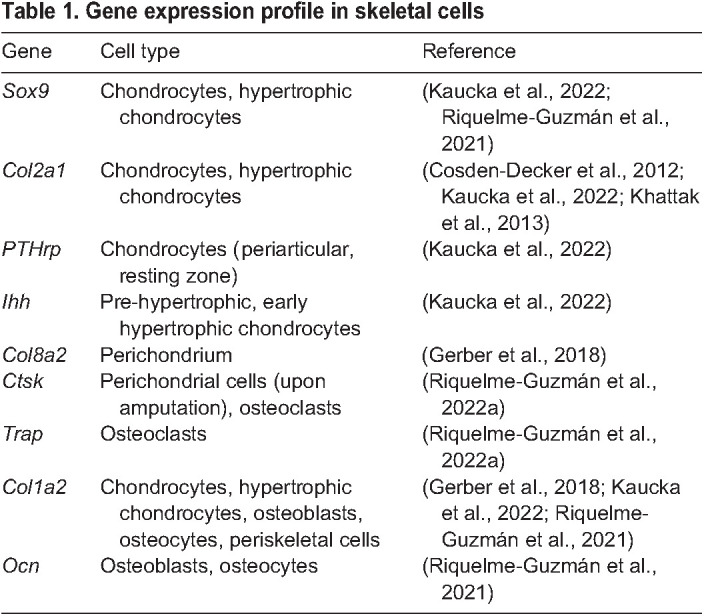
Gene expression profile in skeletal cells

During endochondral ossification, hypertrophic chondrocytes can transdifferentiate into bone cells and marrow adipocytes (Giovannone et al., 2019; Jing et al., 2017; Yang et al., 2014). The participation of cartilage cells in ossification and formation of marrow adipocytes was recently shown in axolotls (Riquelme-Guzmán et al., 2021); however, whether these are hypertrophic chondrocytes remains unknown. Additionally, a hybrid cell type was reported close to the chondro-osseous junction expressing both SOX9 and osteocalcin. These observations could potentially demonstrate a plastic cellular state of hypertrophic chondrocytes in the axolotl and also their participation in osteogenesis. Similar plasticity was reported in newts, where some hypertrophic chondrocytes in distal lacunae, close to the erosion bay, synthesise bone-like collagen fibrils (Quilhac et al., 2014).

Recently published scRNA-seq datasets contain several skeleton-related populations and their most transcribed genes (Gerber et al., 2018; Leigh et al., 2018). Albeit these datasets do not provide exact spatial location of the cells sequenced, they are an extremely useful tool to uncover their transcriptional identity. Besides hypertrophic chondrocytes, two important populations to consider are perichondrial and periosteal cells, i.e. cells located in the periphery of cartilage or bone, respectively, which play a major role during regeneration. The specific molecular signature of these cells in salamanders remains undefined, as well as their role during ossification. In axolotls, several highly expressed transcripts were identified in perichondrial cells based on the expression of *Col8a2* (Gerber et al., 2018). Additionally, lineage tracing using transgenic reporter lines identified that *Col1a2^+^* perichondrial cells contribute to the regenerated structure (Gerber et al., 2018), and that some perichondrial cells express *Ctsk* upon amputation (Riquelme-Guzmán et al., 2022a). Moreover, the expression of *PTHrP* and *Gli1* was reported in the perichondrium of newts (Kaucka et al., 2022). Despite these reports, no unique marker for the perichondrium has been identified and the identity of the periosteum in salamanders remains unexplored. In mouse, many markers have been used to identify periosteal cell populations during bone regeneration, including *Ctsk*, *αSMA*, *Sox9*; however, none of these markers is completely specific for the periosteum (Debnath et al., 2018; Kuwahara et al., 2019; Matthews et al., 2021; Perrin and Colnot, 2022). It is important to note that the term ‘periskeletal cells’ is commonly used in the literature to refer to both perichondrial and periosteal populations given that their similarities and differences still remain to be resolved. We expect future works to address the identity of periskeletal cells as they play a critical role during limb regeneration.

## How is the limb skeleton regenerated in salamanders?

**Fig. 3.**
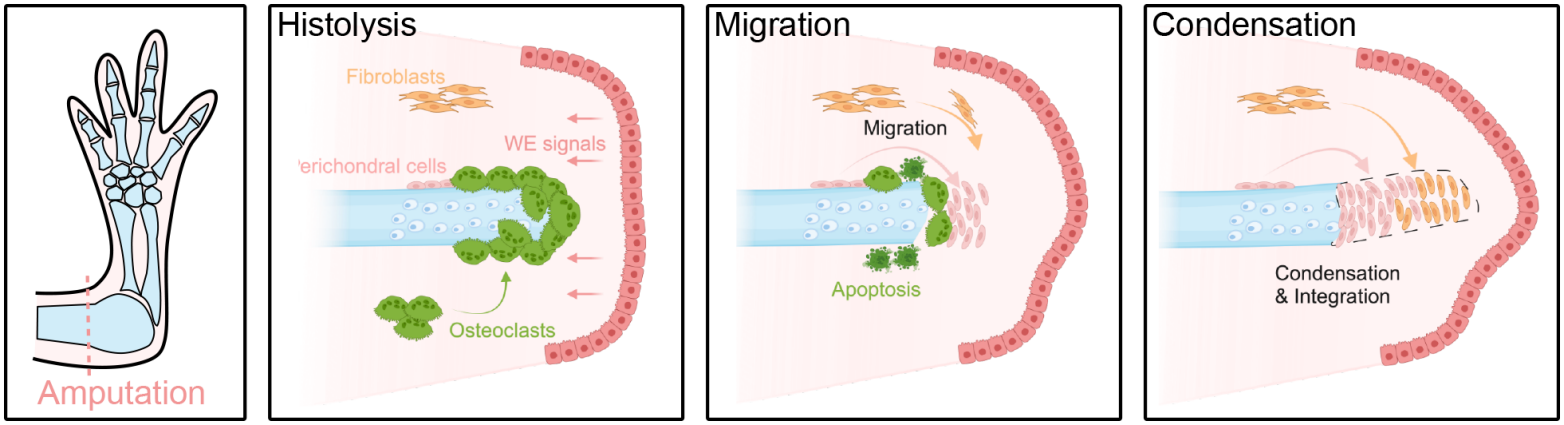
**Cartilage regeneration upon salamander limb amputation: The process of cartilage regeneration in juvenile axolotls starts with the remodelling and histolysis of non-functional tissues.** Signals from the WE induce an inflammatory response and promote skeletal resorption by osteoclasts. Simultaneously, migration of skeletal progenitors from the perichondrium and interstitial space results in the condensation of the new skeleton by differentiation into cartilage cells. Contribution of these progenitors is differential along the proximo-distal axis. The success of the integration of the newly formed cartilage is influenced by skeletal resorption. Created with BioRender.com.

Limb regeneration is successfully achieved by the correct coordination of overlapping phases and events, including wound closure and the formation of a blastema. The appendicular skeleton belongs to the group of connective tissues found along the limb, which collectively play a major role in this process. Among the different salamander species, the axolotl has been the most used model for skeletal regeneration. Here, we summarise the mechanisms involved with skeletal regeneration and present some unresolved challenges lying ahead (Fig. 3).

### First step: wound closure and histolysis

The formation of a wound epithelium (WE) is one of the earliest events during regeneration and is fundamental for wound closure. The WE is a specialised structure, formed by migration of resident keratinocytes flanking the injury site (Hay and Fischman, 1961; Repesh and Oberpriller, 1978), which releases factors necessary for blastema proliferation and patterning (Boilly and Albert, 1990; Ghosh et al., 2008; Han et al., 2001), inflammation, extracellular matrix (ECM) remodelling and tissue histolysis (Tsai et al., 2020).

Tissue histolysis is the clearance of debris and non-functional tissue prior to blastema formation (Stocum, 2017). In the skeleton, a rapid and substantial resorption of the calcified tissue (in both cartilage and bone) occurs starting at 5 days post amputation (dpa) (Riquelme-Guzmán et al., 2022a). This resorption is driven by multinucleated *Ctsk^+^/Trap^+^* resorbing cells. The WE appears to be linked to resorption induction, as blocking its formation by full skin flap surgery downregulates genes associated with ECM remodelling (e.g. *Mmp2*, *Mmp13*, *Ctsk*, among others), inhibits skeletal resorption and reduces number of *Ctsk^+^* cells (Riquelme-Guzmán et al., 2022a; Tsai et al., 2020).

Various studies identified these regeneration-induced resorbing cells as osteoclasts by their multinucleation and/or their expression of *Ctsk* and *Trap* (Fischman and Hay, 1962; Riquelme-Guzmán et al., 2022a; Tank et al., 1976). Osteoclasts are myeloid-derived cells specialised in the degradation of skeletal matrices. A rapid increase in myeloid chemotactic molecules occurs at 1 dpa (e.g. CCL4, CXCL12) followed by an infiltration of myeloid cells and macrophages (Godwin et al., 2013). Blastema cells also promote myeloid cell recruitment by the release of interleukin 8 (Tsai et al., 2019). The increase of chemotactic molecules and infiltration of myeloid cells are likely related to the rapid osteoclast differentiation observed upon amputation. However, it is unknown what molecular mechanisms are involved in the recruitment and differentiation of these cells, and what is the participation and function of other immune cells, if any, in the skeleton.

### Second step: skeletal progenitor cells and blastema formation

During salamander limb regeneration, mature tissues provide progenitor cells with lineage-restricted regenerative capabilities (Kragl et al., 2009). Due to the multi-tissue composition of the limb, several works have specifically focused on the participation of skeletal tissues in regeneration and the origin of skeletal progenitors. Early grafting experiments showed that dermal cells contributed to skeletal regeneration (Dunis and Namenwirth, 1977) and only a reduced number of cells found inside the skeleton contributed to the blastema (Muneoka et al., 1986). Later works using grafting of GFP^+^ tissues, transgenesis and *in vivo* confocal imaging demonstrated that cells embedded in the cartilage or bone do not participate in regeneration; instead, regeneration of the skeleton is carried out by periskeletal cells, dermal and interstitial fibroblasts (Currie et al., 2016; Gerber et al., 2018; McCusker et al., 2016). These cell populations contribute sequentially along the proximo-distal axis to the regenerated skeleton; periskeletal cells regenerate the most proximal regions and are strongly associated with formation of a cartilaginous callus, while fibroblasts are the main contributors to the distal regenerate, but still participate in proximal regeneration (Currie et al., 2016; Gerber et al., 2018). The cartilaginous callus corresponds to the condensation of progenitors wrapping the amputated element (Kaucka et al., 2022), and represents the first point where the missing tissue starts to re-grow.

The mobilization of skeletal progenitors is associated with tissue histolysis (Thornton, 1938a,b), an association evidenced by an overlap between skeletal resorption and cartilage condensation (Riquelme-Guzmán et al., 2022a). This coordination of resorption with condensation could point towards an influence of skeletal degradation in the activation or migration of skeletal progenitors. In bone homeostasis, a crosstalk between osteoclasts and osteoblasts has been demonstrated, influencing both bone degradation and formation (Furuya et al., 2018; Ikebuchi et al., 2018). Surprisingly, the accumulation of skeletal progenitors after resorption occurs below the amputation plane. The stump likely collapses due to a lack of physical support produced by skeletal degradation. When resorption is taken into account, there is a disparity between the amputation plane and the location of blastema formation, a feature already observed in another regeneration model: the mouse digit tip (Seifert and Muneoka, 2018).

The molecular signature of skeletal progenitors is still unknown. Connective tissue cells generally produce the PRRX1 protein (Gerber et al., 2018; Satoh et al., 2010a) and require PDGF-BB signalling for migrating into the blastema (Currie et al., 2016). Additionally, interleukin 8 induces proliferation of perichondrial cells in intact limbs (Tsai et al., 2019). As connective tissue-derived blastema cells reach a relative transcriptionally homogeneous state before re-differentiation, the tissue of origin of fibroblast contributing to the distal skeleton could be diverse and will require further investigations. As a comparison, in mice, fibroblasts surrounding peripheral nerves or skeletal muscle contribute to bone regeneration (Carr et al., 2019) or bone repair, respectively (Julien et al., 2021). Despite this transcriptional homogeneity of connective tissue-derived blastema cells, periskeletal cells only contribute to the proximal skeleton in axolotls. However, we still do not know the molecular profile of the perichondrium and periosteum in salamanders, and how they might participate, possibly differentially, during cartilage and bone regeneration.

Remarkably, when isolated periosteum was transplanted into a limb 48 h post amputation, periosteal cells contributed to various connective tissues in the regenerated limb, such as the skeleton and the connective tissue associated with muscle and blood vessels (McCusker et al., 2016). Periosteal cells thus have the capacity to regenerate other connective tissues, but their natural association with the skeleton directs their regenerative potential solely towards skeletal tissues. Many mechanisms could be influencing this outcome, such as their interaction with skeletal cells or ECM, which could hinder their response to de-differentiation or re-differentiation cues provided by the regenerative environment.

### Third step: condensation and integration of the new skeleton

The migrating skeletal progenitor cells condensate forming the new skeletal element. In animals with cartilaginous appendicular skeleton, this condensation results in the formation of the missing cartilage and the ensuing calcification of their diaphyses as the animals mature (Currie et al., 2016; Gerber et al., 2018; Riquelme-Guzmán et al., 2022a). On the other hand, in ossified limbs progenitor cells condensate, first forming a cartilage anlage (Kaucka et al., 2022; Stock et al., 2003; Tank et al., 1976). The ossification of these elements starts during the late stages of regeneration, when the limb has reached a similar size as the contralateral and does not contribute to the extension of the length of the regenerated limb (Kaucka et al., 2022). Moreover, regeneration-induced ossification of the amputated element is delayed compared to the more proximal elements, which are formed *de novo* (Stock et al., 2003). A few markers have been evaluated during the condensation of the regenerated cartilage. Expression of the transcription factor *Runx2* was detected distally to the regenerating cartilage expressing collagen type II (Hutchison et al., 2007). Additionally, *PTHrP*/*Ihh* signalling pathway is dynamically expressed in the cartilage anlage, with a radial arrangement at the beginning of condensation, leading to a proximo-distal expression later on (Kaucka et al., 2022).

Several skeletal anomalies have been reported during limb regeneration. When amputations due to conspecific bites occur, 80% of larvae and 50% of adults present defects in the regenerated skeleton (Thompson et al., 2014). Upon surgical amputation, fractures at the level of amputation, constrictions of the elements, higher bone volume, disorganised collagen fibres at the mature-regenerated interphase (i.e. where the regenerate integrates to the stump tissue) and deficient integration of the regenerate have been reported (Bothe et al., 2021; Kaucka et al., 2022; Riquelme-Guzmán et al., 2022a). These defects seem to be influenced by the intrinsic mechanisms of regeneration. The efficiency of regeneration is affected by skeletal resorption (Riquelme-Guzmán et al., 2022a), showing that histolysis is important for priming the skeleton for its integration. Indeed, Tsutsumi et al. hypothesised that changes in the ECM of the distal humerus at the amputation plane helped the regeneration and integration of the joint to the stump (Tsutsumi et al., 2015). Resorption has also been linked to a bulkier bone phenotype (Riquelme-Guzmán et al., 2022a), which results from a continuous radial expansion of the cartilage prior to its ossification (Kaucka et al., 2022).

While many unknowns remain behind the mechanisms involved in tissue integration and the frequency of the anomalies reported, vitamin D and retinoic acid have been identified as signalling molecules involved in skeletal regeneration (Nguyen et al., 2017; Vieira et al., 2018). Particularly, retinoic acid might influence osteoclasts numbers, skeletal resorption and chondrocyte differentiation, while vitamin D has an effect on tissue integration by an unknown mechanism. Both retinoic acid and vitamin D influence pattern formation during regeneration, and it was recently shown that positional identity could play a role in a seamless integration using the accessory limb model (ALM) in axolotls (Vieira et al., 2023). The ALM has been useful to understand that patterning and differentiation of the regenerated skeleton is independent of the template; however, the formation of the ALM is not integrated to the limb skeleton unless the skeletal tissue is injured (Endo et al., 2004; Satoh et al., 2010b), which further demonstrates the requirement of skeletal cells for the regeneration of the proximal skeleton and suggests a crucial role of these cells in the integration of the regenerate.

## Amputation-independent appendicular skeleton regeneration

Fractures are the most common skeletal injuries in humans. Generally speaking, the repair of bone injuries entails three major phases: inflammation, endochondral bone formation and coupled remodelling. A cartilage callus bridges the gap in between the fractured element, and this intermediate is later ossified and remodelled by several rounds of bone resorption and deposition (Einhorn and Gerstenfeld, 2015). Notably, fractures can also be repaired by direct bone formation (intramembranous ossification), especially in cases where the fractured element is firmly stabilised (Thompson et al., 2002). Given its medical relevance, fracture healing has gained more attention in the salamander field.

Several works have assessed fracture regeneration in axolotls. Non-stabilised union fractures are regenerated in over 5 months through the formation of a cartilaginous callus (Hutchison et al., 2007; Mitogawa et al., 2015). Resections of articular cartilage also regenerate in 5–6 months, with formation of callus-like structure as early as 2–4 weeks after the injury (Cosden et al., 2011; Lee and Gardiner, 2012). On the other hand, large bone resections known as critical size defects (CSD) are not regenerated (Chen et al., 2015; Cosden-Decker et al., 2012; Hutchison et al., 2007; Polikarpova et al., 2022; Satoh et al., 2010a). CSD are the smallest fracture that will result in no spontaneous regeneration, hence resulting in the need for a surgical intervention (Vajgel et al., 2014). Noteworthy, most of these fracture studies were performed with animals of various sizes and ages, for example: Hutchison et al. studied 4 mm radius or tibia CSD in 3–5 cm axolotls; Satoh et al. did 2 mm radius CSD in 8–12 cm axolotls; and Cosden-Decker et al. performed 4 mm tibia CSD in 7–12-month-old axolotls, without stating the size. This variance makes it difficult to draw comparisons, particularly when considering the relative dimensions of the CSD or the skeleton developmental phase, as in some cases the cartilage was fractured and not bone.

A recent study proposed a standardised methodology for assessing fracture healing in adult axolotls and comparing it to the existing mouse models (Polikarpova et al., 2022). In this work, fractures in fully ossified femurs formed a callus after 3 months and regeneration was almost complete after 9 months. A proliferative population surrounding the cartilaginous callus seems to participate in the repair, together with an accumulation of SOX9^+^ cells. This SOX9^+^ population remains until 6 months post injury, and its clearance was correlated with the ossification of the callus. In mice, SOX9^+^ periosteal cells participate in fracture healing giving rise to chondrocytes, osteoblasts and osteocytes. Remarkably, upon rib fracture, the presence of a hybrid osteochondral cell, co-expressing cartilage and bone markers, was reported (He et al., 2017; Kuwahara et al., 2019). In axolotls, similar hybrid cells participate during ossification and fracture healing of appendicular long bones (Mitogawa et al., 2015; Riquelme-Guzmán et al., 2021).

Given the intrinsic regenerative capacities of axolotls, attempts to stimulate regeneration of CSD have been pursued. Using scaffolds soaked with BMP4+HGF or a whole tissue extract, regeneration of 30% of CSD defects in the fibula was achieved (Chen et al., 2015). Cosden-Decker et al. transplanted tissue from the joint into a 4 mm CSD in the tibia and reported a successful bridge between the extremes of the element, although the regenerate lacked bone formation and rather an ectopic joint formed 7 months later (Cosden-Decker et al., 2012). Interestingly, transplantation of blastema cells led to integration and regeneration of 60% of CSD in radius. These cells differentiate into chondrocytes expressing collagen type II (Satoh et al., 2010a), suggesting that the fracture environment is permissive for regeneration but fails to induce a substantial number of regenerative-competent cells. This contrasts to regeneration upon amputation, where a whole-limb (and possibly a whole-body) response is triggered, and skeletal progenitors are recruited from other connective tissues. It is important to bear in mind that a fragment resection that regenerates require a proximal and a distal point of tissue integration, and histological evidence from the previous works shows a poor mature-regenerate transition. Clearly, a better understating of successful fracture healing would shed lights on its failure during CSD. The identity of progenitor cells remains a cornerstone of regeneration, but earlier events, such as tissue inflammation, and later events, such as tissue remodelling and integration, will also require further investigations.

## New research opportunities

Whether it is an amputation or a fracture, the salamander skeleton possesses an efficient toolkit for regeneration. Comparisons between mechanisms activated by different injuries provide an opportunity to understand how environmental signals and the intrinsic cellular states influence the outcome of regeneration. Noteworthy, the heterogeneity of the tissue across the proximo-distal axis has to be considered, as the skeletal elements are composed of cartilage, bone and a marrow cavity. This variable cellular landscape might directly influence the regenerative response. For example, in skeletal elements of adult axolotls, regeneration of an amputation at the mid-diaphysis might be different to an amputation across the epiphysis. This heterogeneity could determine the recruitment and differentiation of immune cells regulating resorption, or the recruitment of various progenitor cells arising from the periosteum, perichondrium or both. To comprehensively understand the formation and integration of new skeleton, it will be important to take into account the cellular landscape, age and size of animals used for each study.

In addition to the injury site and cellular components, a key aspect of regeneration is tissue integration, which is fundamental for the functional re-establishment of the limb, and it must occur in every tissue within. Both resorption and positional identity were shown to influence integration; however, our understanding of this topic is still a work in progress. Key research gaps to explore include how the ECM is remodelled to function as a template for condensation of the new skeleton, and how (and if) cells at the mature-regenerated interphase communicate in order to seamlessly bind the two structures and direct re-differentiation of skeletal progenitors. Additionally, studying periskeletal cells, the first line responders, will also offer clues on how a seamless transition is orchestrated.

Regardless of the many challenges lying ahead, the increasing availability of new technologies are enabling the exploration of different aspects of skeletal regeneration. Salamanders are unique model organisms useful for performing in-depth studies using, for example, advanced optical microscopes (e.g. two-photon microscopy) or grafting of new biomaterials. Recent works assessing tissue mechanical properties and pairing quantifications with predictive computational models (Comellas et al., 2022; Kondiboyina et al., 2024; Riquelme-Guzmán et al., 2022b) demonstrate their unique potential. We are hopeful the field will continue to move forward with studies tackling the most pressing issues. Ultimately, salamanders have proven to be useful models for tackling skeletal regeneration with a direct potential to benefit human medical research.
